# Investigation of Short Chain PFAS Degradation Efficiency Using Free-Standing Boron Doped Diamond Electrodes at High Current Density in a Flow Cell

**DOI:** 10.1021/acselectrochem.5c00121

**Published:** 2025-08-12

**Authors:** Marius Amerio-Cox, Joshua J. Tully, Fengping Tang, Anna Dettlaff, Julie V. Macpherson, Timothy Mollart, Tim Sidnell, Simone Mathias, Patrick Sears, Madeleine J. Bussemaker

**Affiliations:** a School of Chemistry and Chemical Engineering, 3660 University of Surrey, Guildford GU2 7XH, U.K.; b Department of Chemistry, 2707 University of Warwick, Gibbet Hill Road, Coventry CV4 7AL, U.K.; c Faculty of Chemistry, 49557 Gdańsk University of Technology, 11/12 Gabriela Narutowicza Street, 80-233 Gdańsk, Poland; d Element Six (UK) Limited, Oxford, OX11 0QR, U.K.

**Keywords:** PFBS, PFBA, boron doped diamond (BDD), free-standing BDD, electrochemical oxidation, high current density, flow cell, bipolar electrodes, defluorination efficiency, fluoride mass balance, LC-MS/MS analysis

## Abstract

Short chain PFAS is known to be more challenging to destructively remove than its longer chain counterparts. Electrochemical oxidation at boron doped diamond (BDD) electrodes is one promising way forward. The majority of investigations are carried out using thin film, high grain density BDD electrodes (attached to the growth substrate) at low current densities of <50 mA cm^–2^. In this work, the impact of high current density on short chain (C_4_), perfluorobutanoic acid (PFBA), and perfluorobutanesulfonic acid (PFBS) removal rates and defluorination efficiency is investigated in a recirculating flow system. These studies are carried out using free-standing BDD electrodes, which are grown thick enough so that the BDD can be removed from the non-diamond growth substrate and thus contain a much lower grain density compared to thin-film BDD. The cell utilizes four BDD electrodes, where only the two outer electrodes are directly connected to a potential supply, the two inner electrodes are electrically unconnected, and driven in a bipolar arrangement. Solutions contain saturated potassium sulfate as the electrolyte. A current density of ≥390 mA cm^–2^ (after correcting for surface roughness) is employed for times up to 9 h. PFBS/PFBA concentrations in the range range ∼1–60 mg L^–1^ are investigated. Importantly when comparing rate constant data to literature for similar concentrations (after normalization of the rate constants to treatment volume/anode area), the values suggest removal rates approximately an order of magnitude higher than those at lower current density (in stirred solutions). Defluorination efficiency is also found to be higher with (close to) complete defluorination indicated at higher concentrations/longer times for short chain PFAS. Microscopy analysis of the free-standing electrodes after deployment for >90 h of advanced oxidation reveals no obvious signs of corrosion. This likely reflects both the reduced grain boundary density and lower sp^2^ carbon content in thick free-standing BDD. The data highlight the potential for this electrode material in long term electrochemical treatment of short chain PFAS solutions in recirculating flow systems.

## Graphical abstract

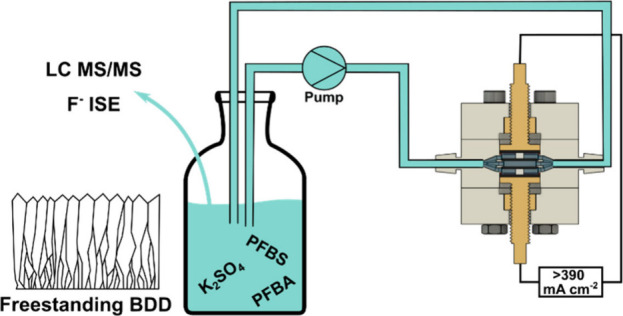

## Introduction

Per- and polyfluoroalkyl substances (PFAS) constitute a group of anthropogenic compounds extensively used in industrial, commercial, and consumer applications since the 1950s. PFAS are chemically and thermally stable, making them suitable for a broad range of applications such as firefighting foams, textiles, nonstick coatings, and food packaging.^1^ The widespread use of PFAS has resulted in ubiquitous environmental contamination, especially in water sources, and no natural pathway for their degradation exists.^2,3^ While manufacturing of the C_8_ PFAS species perfluorooctanoic acid and perfluorooctanesulfonic acid is largely phased out across the world, this has resulted in an increase in the manufacture of shorter chain PFAS (<C_7_).^4^ Short-chain PFAS are also products of long-chain degradation.^4^

Several destructive technologies are being developed for PFAS treatment in water systems including ultrasound,^5,6^ photocatalysis,^7^ chemical oxidation,^8^ and electrochemical oxidation.^9^ All can be combined with separation/concentration stages such as adsorption, filtration, fractionation, and reverse osmosis, resulting in lower volume waste streams for destructive treatment. However, achieving breakdown of short chain PFAS such that complete defluorination results is challenging.^4,10–12^ Electrochemical oxidation approaches have demonstrated significant potential for PFAS remediation due to their simplicity, ease of operation and efficiency.^9^ A large body of research has focused on PFAS electrochemical degradation using boron doped diamond (BDD) electrodes.^9^ BDD is described as a “nonactive” electrode which favors production of (weakly adsorbed) hydroxyl radicals (^•^OH) from water oxidation.^13,14^ The mechanical and chemical robustness of BDD also greatly reduces susceptibility to corrosion when subject to high anodic potentials.^15^ PFAS destruction is thought to occur first via direct oxidative electron transfer (rate limiting step),^16^ followed by attack from highly oxidizing radicals, which are generated electrochemically.^17^ These include ^•^OH but also radical anions generated from oxidation of electrolyte anions, such as the sulfate radical, SO_4_^•–^, from SO_4_^2–^.^18^ Mechanistic schemes for the electrochemical breakdown of PFAS are given in ref 9.

BDD electrodes are grown in polycrystalline form using either hot filament or microwave chemical vapor deposition (CVD) on growth substrates which can withstand the high temperatures of CVD growth.^19^ The quality of the BDD is very much dependent on the growth conditions, and reported studies vary in terms of boron dopant density, sp^2^ carbon (non-diamond carbon) content, grain size (density), and morphology.^19–21^ To the best of our knowledge, all PFAS investigations, bar one which uses free-standing BDD,^11^ employ thin film BDD still attached to the growth substrate. When grown as a thin film, the grains are in the micro- or nanocrystalline regime.^22^ The smaller the grain, typically the higher is the reported sp^2^ carbon content; this is especially true of nanocrystalline material, due to the prevalence of sp^2^ carbon at grain boundaries. sp^2^ carbon incorporation also promotes different water oxidation routes (water oxidation is kinetically more facile on sp^2^ carbon),^19^ makes the material much more susceptible to anodic corrosion, and decreases mineralization efficiency.^23^ Nanocrystalline BDD was shown to remove PFAS at much slower rates compared to microcrystalline BDD, attributed in part to the presence of sp^2^ carbon.^22^

If CVD diamond is grown thick enough, ∼ 0.4 mm minimum thickness (using microwave techniques), the diamond can be removed from the growth substrate with its structural integrity maintained and used as a free-standing material. The synthesis technology for free-standing CVD diamond is already a mature and high-volume production market.^24^ Such manufacturing processes can be adapted to grow free-standing BDD electrodes^19^ for commercial uses^25^ (up to ∼180 mm in diameter),^26^ and the growth substrate can also be recycled or reused for CVD growth. For electrochemical oxidation applications, the increased thickness results in larger grains, leading to a reduced density of grain boundaries and reduced amounts of sp^2^ carbon in the surface.^15^ The thicker free-standing material also dictates the electrodes will last longer in the field, and there are no concerns about chemical stability of the growth substrate. Such electrodes should also be able to tolerate higher current densities for longer periods of time than thin film BDD. Free-standing electrodes can also easily be used in a bipolar configuration, where both faces function as electrodes.

Most electro-oxidation studies use a current (expressed as current density) rather than a voltage to control the PFAS electrochemical removal rate (via oxidative breakdown). Typically, thin film BDD electrodes of areas 25–140 cm^2^ and current densities of 0.04–50 mA cm^–2^ (currents of ∼0.1–0.8 A) are employed; see PFAS summary tables in the supporting information associated with refs 9 and 22. In the electrolyte system, many different processes contribute to the anodic current. These include water oxidation, PFAS oxidation, and oxidation of electrolyte anions. While increasing current density is effective at increasing the destruction rate of longer chain PFAS (C_6_ and above),^27–29^ there are minimal studies which look at the impact of a high current density on the removal rate and defluorination efficiency of shorter chain analogues. Previous work using thin film BDD has found suboptimal defluorination efficiencies for short chain PFAS removal using BDD electrodes at the lower current densities.^12,30,31^ Interestingly, one study found that raising the current density (up to 200 mA cm^–2^) in a leachate solution was beneficial towards the destruction of the shorter chain (C_4_ and C_6_) intermediates formed during the degradation of longer chain PFAS.^32^

Given the growing prevalence of shorter chain PFAS, their inherently slower breakdown kinetics, and the need to achieve complete defluorination, this work investigates the use of low sp^2^ carbon content, free-standing BDD electrodes for short-chain PFAS destruction and defluorination. Their effectiveness, in terms of defluorination efficiencies, removal rates, and material stability (after extended operation >90 h), when working under high current density conditions in a recirculating flow cell is also investigated. For this study we use C_4_ PFAS, in particular, perfluorobutanoic acid (PFBA) and perfluoro­butanesulfonic acid (PFBS) in a saturated sulfate solution (∼0.63 M), given sulfate ions are a source of SO_4_^•–^ ^33^ and sulfate has been shown to increase PFAS removal rates in electrochemical oxidation systems.^31^ A flow cell is used to increase mass transfer, and the electrodes are employed in a bipolar arrangement, a BDD anode and cathode with two free-standing BDD electrodes placed between the outer electrodes. The bipolar setup increases the anode area and enables the electrodes to be more closely spaced, which reduces ohmic losses. Both the kinetic rate constants for PFBA and PFBS removal and defluorination efficiency, where 100% represents complete defluorination, are extracted for concentrations of 0.70–66 mg L^–1^ (∼μM to 300 μM), a range associated with PFAS concentrates.^34^

## Experimental Section

### Materials

98% PFBA, 97% PFBS, 98% ammonium acetate, and 0.1 M analytical grade sodium fluoride (NaF) were purchased from Merck. Reagent grade potassium sulfate (K_2_SO_4_) and pH 4 buffer tablets were purchased from Fisher Scientific. K_2_SO_4_ solutions were prepared saturated at ambient temperature (∼20 °C), equating to ∼110 g L^–1^ (∼0.63 M). Liquid chromatography (LC)–mass spectrometry (MS) grade methanol and water were purchased from ROMIL Ltd. Ultrapure (Milli-Q) water was provided by an in-house device operating at 18.2 MΩ cm.

Free-standing BDD electrodes were cut from Diafilm Electrochemical-Processing grade polycrystalline BDD wafers (Element Six, Oxford, U.K.), 0.42 mm thick. The BDD was doped sufficiently such that it was above the metallic threshold (>10^20^ B atoms cm^3^).^19^ The nucleation face was previously in contact with the growth substrate prior to the BDD being removed (Figure 1a). The surface roughness (*S*_*q*_), measured using white light interferometry (WLI), was ∼100 nm for the nucleation face and ∼8 μm for the growth face. The surface area of the growth face was estimated from WLI and electrochemically as described in Supporting Information **1a** and found to be about twice the geometric area. The grain sizes between the two faces are also different. The nucleation face contains smaller grains, typically less than 1 μm in size, and the growth face comprises grains ca. 30 μm in size, Figure 1a.

**Figure 1:**
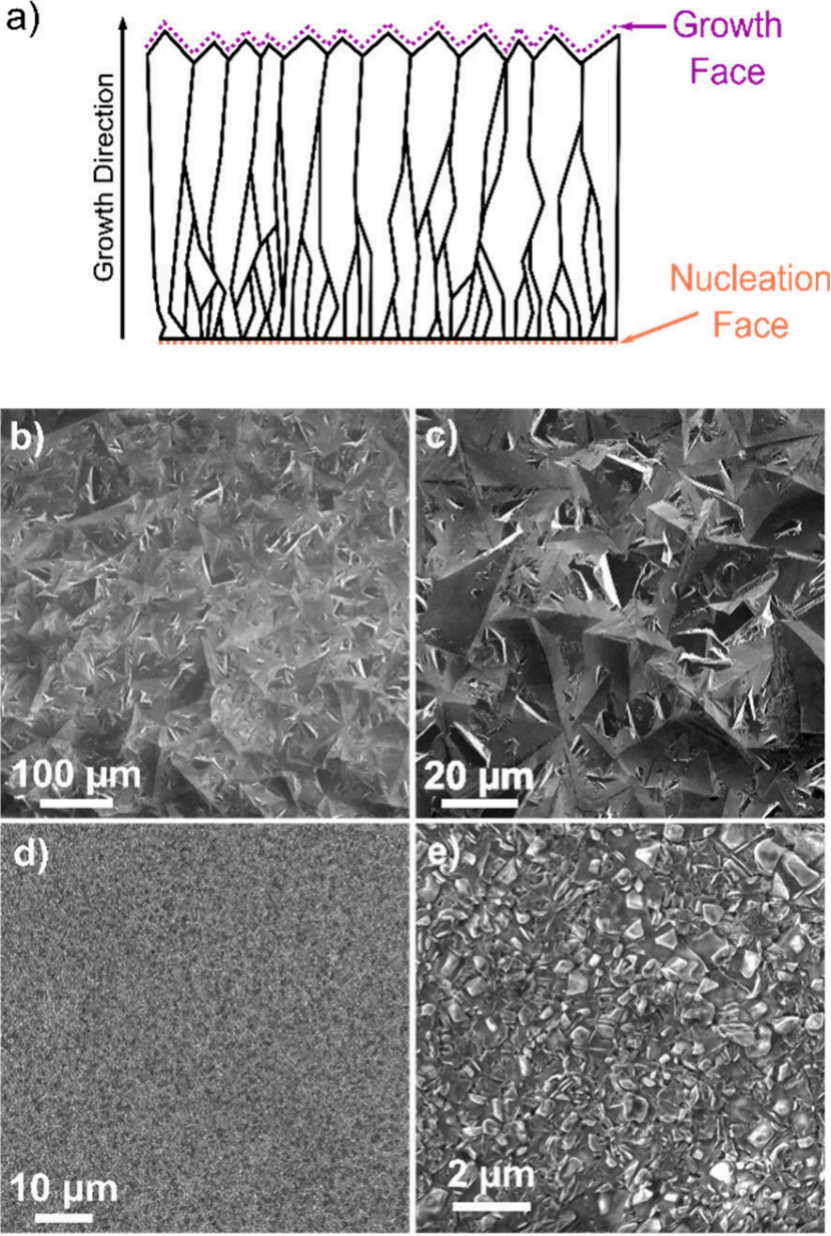
(a) Schematic of the grain structure of the free-standing polycrystalline BDD used in this study, showing the nucleation face (orange) and the growth face (purple). The growth face is always the anode in the electrochemical oxidation experiments. Scanning electron microscopy (SEM) images of the BDD growth (b, c) and nucleation (d, e) face at low (b, d) and higher (c, e) magnification.

### Electrolysis System

The flow cell used in this work was designed in Fusion 360 (Autodesk, U.K.) and 3D printed in polymethyl methacrylate (Clear, Formlabs) on a Form 3 SLA 3D printer. The cell (Figure 2) comprises four BDD circular free-standing electrodes, two outer electrodes 1 cm in diameter (only growth face exposed), and two inner electrodes 0.85 cm in diameter, with both faces exposed. The disk electrodes were cut to size using a laser micromachining system and acid cleaned as described elsewhere.^35^ Acid cleaning oxygen terminates the BDD surface.^19^ For the outer electrodes, the nucleation face is sputtered with an ohmic contact comprised of a trilayer structure of Ti|Pt|Au, 50/50/200 nm thickness, which is in contact with a brass screw and electrically isolated from solution. The brass screw also acts to compress an O-ring, which is used to create a water-tight seal. For the two inner electrodes, the electrodes are held in place by a 2 mm diameter silicone disk. Solution flows around the silicone disks in the cell. For the bipolar electrodes the growth face is always used as the anode (oxidation) as this surface contains significantly reduced sp^2^ carbon content compared to the nucleation face. Raman and electrochemical analyses of the growth surface are shown in Supporting Information section SI.1b.

**Figure 2:**
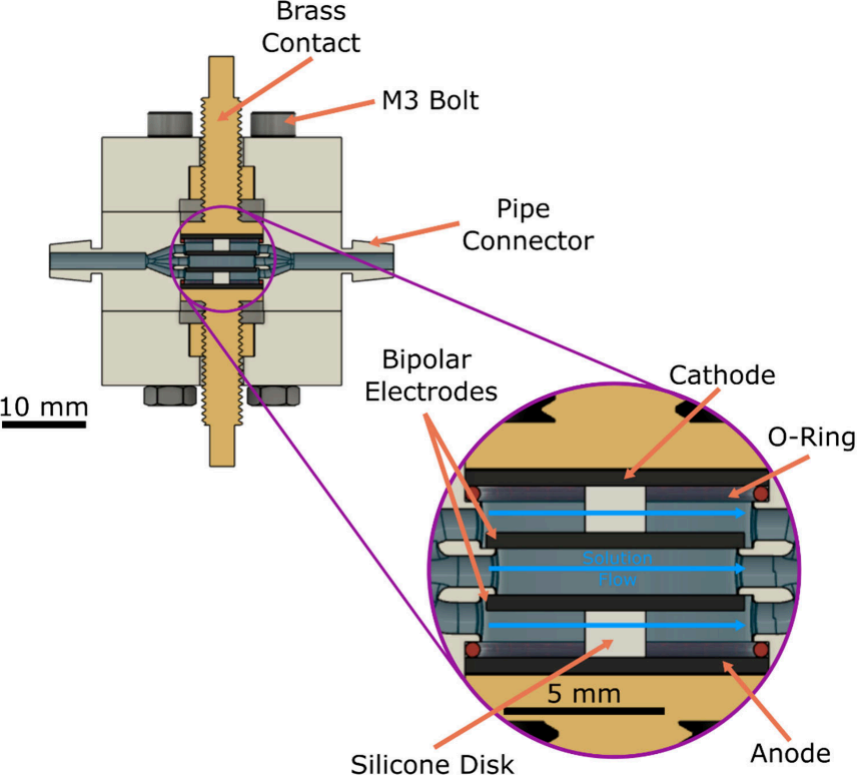
Schematic of the 3D-printed cell used for PFAS electrolysis experiments showing the two monopolar and two bipolar electrodes as well as the flow path and electrical contacts.

Sealing the electrodes in the cell results in a reduction in the surface areas of each electrode face to 0.64 cm^2^ and 0.52 cm^2^ (geometric area) for the outer and inner electrodes, respectively. The electrode–electrode spacing is 1.5 mm. The inner electrodes are driven without a direct electrical connection, in a bipolar fashion. The total geometric anode area of the cell is 1.68 cm^2^ (one outer anode and two inner anodes), which increases to ∼3.36 cm^2^ when surface roughness is accounted for.

During electrolysis, 250 mL of solution was recirculated through the cell at a flow rate of 100 ml min^–1^ via a peristaltic pump (Watson Marlow 313S). A 500 mL bottle was used as a solution reservoir and silicone tubing (3.2 mm internal diameter, 50 mL volume) was used to connect the flow cell to the reservoir via the pump. The bottle was held at 50°C using a temperature-controlled water bath (PolyScience) to minimise any temperature fluctuations arising from Joule heating, which are likely given the high current densities applied. The entire cell was placed in a fume hood due to the evolution of gases during electrolysis. Prior to electrolysis, the electrolyte was pumped through the cell at 100 mL min^–1^ for 20 min to equilibrate the temperature of the system. The cell was operated in galvanostatic (current limited) mode using a benchtop power supply (PSU, Weir 423D) at a constant current output of 0.5 A. This equates to a current density of ∼480 mA cm^–2^ for the inner electrodes and ∼390 mA cm^–2^ for the outer electrodes (using true surface area and not the geometric area) in this bipolar set-up. At predetermined time points a 2.5 mL sample was taken from the electrolyte reservoir and stored at 2–5 °C in glass vials.^36^ The voltage at each timepoint was also recorded to calculate power consumption across the cell.

For these studies, experiments were carried out in potassium sulfate solutions^31,37^ at saturated concentration. At the end of the experiment, the system was pumped to dryness, and the electrolyte fluid volume was recorded to correct for losses through evaporation and/or leaking. The system was then washed for 10 min with 250 mL ultrapure water at a flow rate of 100 mL min^–1^ to remove PFAS which may have adsorbed onto the walls. A 2.5 mL sample was taken for liquid LC-MS/MS analysis. While no PFAS in the water sample was revealed (within the limit of detection), the system was flushed with a further 500 mL of water and 150 mL of methanol as a precaution to minimize carryover between experiments. All other glassware used was washed three times with water and three times with methanol to desorb any remaining PFAS.^38^

Solutions with concentrations in the range ∼1–60 mg L^–1^ were made up and final concentrations determined via methods detailed in Analytical Methods. Specifically, degradation of PFBA was tested at concentrations of 65.7 mg L^–1^ and 19.8 mg L^–1^, for 3 h and at 0.947 mg L^–1^ for 9 h. PFBS was tested at concentrations of 57.2 mg L^–1^ and 30.2 mg L^–1^ for 3 h and 0.703 mg L^–1^ for 9 h. All experiments were carried out in triplicate (*n* = 3).

### Analytical Methods

Quantification of PFAS concentrations throughout each electrolysis was carried out by LC-MS/MS (Waters Acquity UPLC, Waters TQD detector) using electrospray ionisation operated in negative mode.^39^ Further details are found in Supporting Information section SI.2. LC separation was achieved using a Waters BEH C18 1.7 μm, 2.1 mm × 100 mm column with 20 mM ammonium acetate in water and methanol mobile phases. Standards and blanks were matrix matched to the samples using the same batch of saturated potassium sulfate solution used to prepare the samples and stored in polypropylene vials with polyimide caps (ChromatographyDirect).

Information on electrochemical defluorination efficiency is typically obtained by using ion chromatography or ion selective electrodes (ISEs) to measure released fluoride ion (F^–^) concentrations. For our experiments that take place in high electrolyte concentrations, the F^–^ ISE is the most appropriate. A F^–^ ISE probe (Oakton by Cole-Parmer) connected to a conductivity/pH meter (8601 AZ) was used to quantify F^–^ concentration at each time point. A description of the F^–^ ISE method can be found in Supporting Information section SI.3.

### SEM Imaging

Field emission scanning electron microscopy (FE-SEM) was used to image the electrodes using the in-lens detector on a Zeiss Gemini FE-SEM 500 operating at 4 kV.

## Results and Discussion

### High Concentration PFBA and PFBS

The initial electrolysis solutions consisted of 19.8 mg L^–1^ (0.092 mM) and 65.7 mg L^–1^ (0.31 mM) PFBA concentrations and 30.2 mg L^–1^ (0.10 mM) and 57.2 mg L^–1^ (0.19 mM) PFBS concentrations in saturated potassium sulfate solutions (∼0.63 M at 20 °C). The saturated sulfate results in a very conductive media (conductivity = 91 ± 0.05 mS) which also helps to reduce any ohmic losses during electrolysis. At these concentrations, PFBA/PFBS micelle formation is not expected.^40^

Two different concentrations were initially investigated to understand how the starting concentration affected removal rates (by electrochemical oxidation) at high current density over 3 h of electrolysis. A current of 0.5 A, typical of that used in thin film BDD studies, was applied but over smaller electrode areas, resulting in a current density of ∼390 mA cm^–2^ and 480 mA cm^–2^ for the outer and inner electrodes, respectively. The voltage between the two outer electrodes varied between 20 and 23 (±2) V for these experiments. Density functional theory (DFT) calculations predict *E*° values for PFBA and PFBS of 2.96 and 3.71 vs SHE respectively (assuming deprotonated states, *vide infra*).^9^ This means that the voltage difference is more than enough to promote oxidation via direct electron transfer of PFAS at the anode (and subsequent follow-up oxidative electron transfer processes) in the bipolar flow cell. There will also be a significantly high overpotential for production of short-lived SO_4_^•‑^ from sulfate oxidation (*E*° = 2.60 V vs NHE)^41^ and OH^•^ from water oxidation (*E*° = 2.80 V vs NHE)^41,42^ respectively at the electrode surface. Both of these radicals can participate in the oxidative breakdown of the short chain PFAS molecules.

The bulk solution pH was monitored during electrolysis of 19.8 mg L^–1^ PFBA (section SI.4) over a 5 h period. The pH increased rapidly from pH = 5.6 to 11 over 15 mins and then more slowly to pH 12, remaining at this value throughout the experiment. While the cathode produces hydroxide ions via the water reduction reaction, production of protons at the anode is occurring via the water oxidation reaction. The latter also results also in, e.g., dissolved oxygen, OH^•^, etc. with the ratio depending on the kinetics of the different water oxidation pathways on the BDD surface, which is dependent on the electrode properties. The high pH indicates that this process is not balanced in terms of protons/hydroxide ions generated. A likely explanation is that a sizeable proportion of the current passing through the anode goes to sulfate oxidation (given the saturated sulfate solution conditions) an electrochemical oxidation process which does not produce protons.^41^ Note, at this pH, as both PFBA and PFBS have relatively low pK_a_’s,^9^ the PFAS molecules will also be deprotonated both at the start and during the experiment (in bulk solution).

Solution analysis was carried out every hour, and the concentration of PFBA and F^–^ was determined using LC-MS/MS and F^–^ ISE respectively (sections SI.2 and SI.3). Figure 3a shows the concentration decrease for both 19.8 mg (blue) and 65.7 mg L^–1^ (purple) PFBA, as a function of time (*n* = 3). Over a 3 h period, 58% and 54% of 19.8 and 65.7 mg L^–1^ PFBA respectively were degraded.

**Figure 3:**
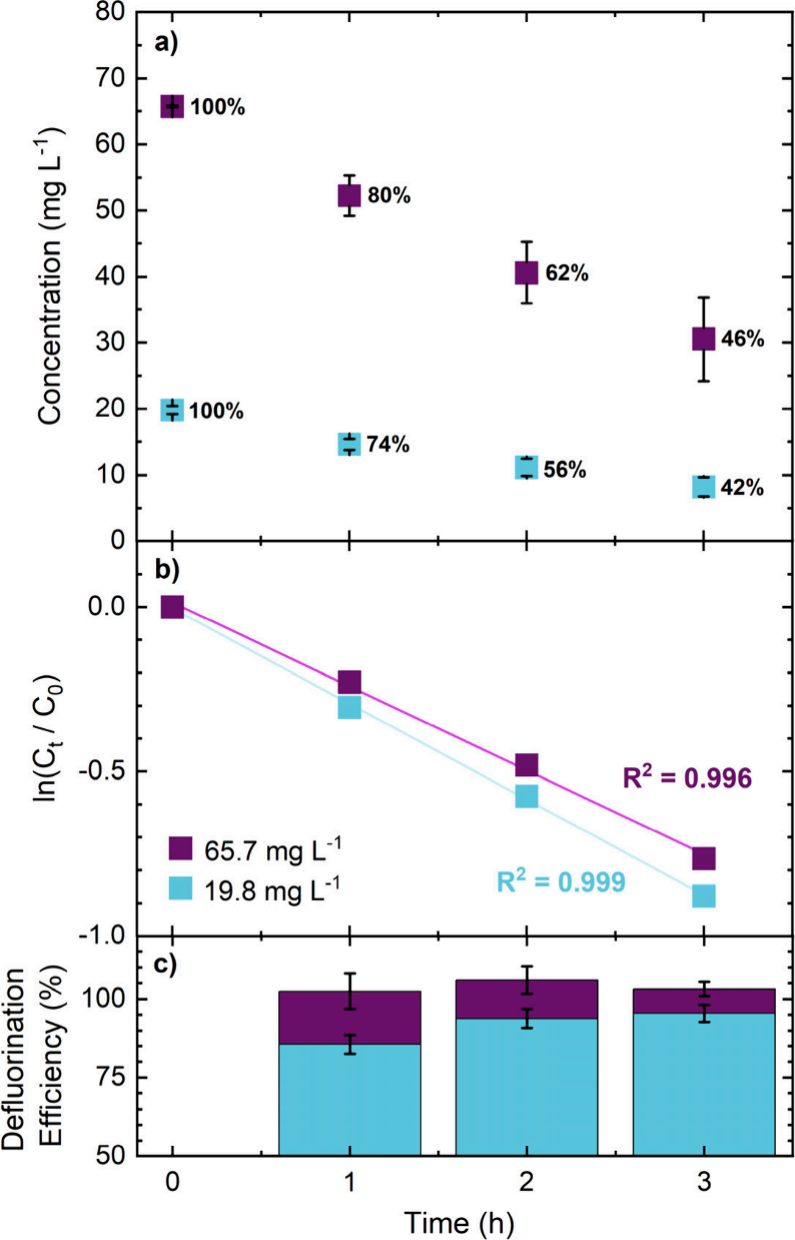
(a) Concentration–time data for PFBA removal at 65.7 mg L^–1^ (purple squares) and 19.8 mg L^–1^ (blue squares) in saturated potassium sulfate solution. The error bars represent the standard deviation (*n* = 3). Current density = 390 and 480 mA cm^–2^ for the outer and inner electrodes respectively; solution volume = 250 mL; total anode area, ∼3.36 cm^2^ (accounting for surface roughness). Numbers next to points represent the proportion of the initial concentration remaining at each time point. (b) First order kinetic plots for the degradation in (a). (c) Bar chart showing the defluorination efficiency (mass balance) in % for each time point.

By plotting ln­(initial concentration of PFAS, PFAS_0_)/(the average concentration of PFAS at time *t*, PFAS_*t*_) versus *t*, a linear relationship was observed indicating a first order reaction for this time period for both concentrations, Figure 3b. A rate constant (*k*) was extracted using eq 1.

$$k=\frac{\ln\left(\frac{{\mathrm{PFAS}}_{0}}{{\mathrm{PFAS}}_{t}}\right)}{t}$$ 1

First order rate constants of 0.29 ± 0.01 (*R* ^2^ = 0.999) and 0.26 ± 0.01 h^–1^ (*R* ^2^ = 0.996) were obtained for 19.8 and 65.7 mg L^–1^ PFBA, respectively (Figure 3b). Interestingly, at the highest concentration, the data was found to fit equally well to zero-order kinetics (*R* ^2^ = 0.996). While interpretation of reaction order requires a quantitative understanding of mass transport in the flow cell, this mixed behavior could be indicative of active surface sites close to saturating at this concentration.^43^ Significant gas evolution was noted (section SI.5) in the outflow pipe, most likely hydrogen and oxygen, which acts to increase turbulence in the system and could also contribute to increasing mass transport in the system. The flowing solution also removes gas bubbles from the surface, helping to free temporarily blocked surface sites. Bubble formation can also be useful in disrupting/preventing any product build-up on the electrode surface. The variations in PFBA concentration for repeat runs may be indicative of small variations in active surface area between measurements.

Figure 3c shows the corresponding defluorination efficiency (fluoride mass balance) for each time point for the two concentrations of PFBA investigated calculated (in %) using eq 2:

$$\mathrm{defluorination efficiency}\left(\%\right)=\frac{{\mathrm{measured moles F}}^{-}}{{\mathrm{theoretical moles F}}^{-}}\times 100$$ 2

The theoretical number of moles of F^–^ produced per time point is calculated by determining the maximum concentration of F^–^ that would result based on the concentration of PFAS that has been removed, as determined by LC-MS/MS, i.e., PFAS_0_ – PFAS_*t*_. Importantly, defluorination efficiency is high (> 85%) for both concentrations at all-time points. For the higher concentration (65.7 mg L^–1^) the defluorination efficiency is very close to 100% at all time points. Values for defluorination efficiency less than 100% suggest that some of the F^–^ is lost as gaseous products, such as F_2_, CF_4_, or C_2_F_6_ or that the F is still covalently bonded to soluble intermediates; albeit small in this case.^44,45^ The high pH of the electrolyte throughout the experiment (section SI.4) means that none of the F^–^ is released as undetectable HF.

The electrochemical degradation of PFBS was trialed under the same experimental conditions with starting concentrations of 30.2 and 57.2 mg L^–1^. Figure 4a shows the reduction in the PFBS concentration with time. The PFBS concentration falls to 33% and 30% of its starting concentration for 30.2 and 57.2 mg L^–1^ PFBS, respectively, over 3 h. The concentration data in Figure 4 was analyzed in terms of eq 1, and first order rate constants for 30.2 and 57.2 mg L^–1^ PFBS of 0.41 ± 0.02 h^–1^ (*R* ^2^ = 0.996) and 0.36 ± 0.02 h^–1^ (*R* ^2^ = 0.994) were determined, respectively. These values are only slightly higher than those observed for PFBA.

**Figure 4:**
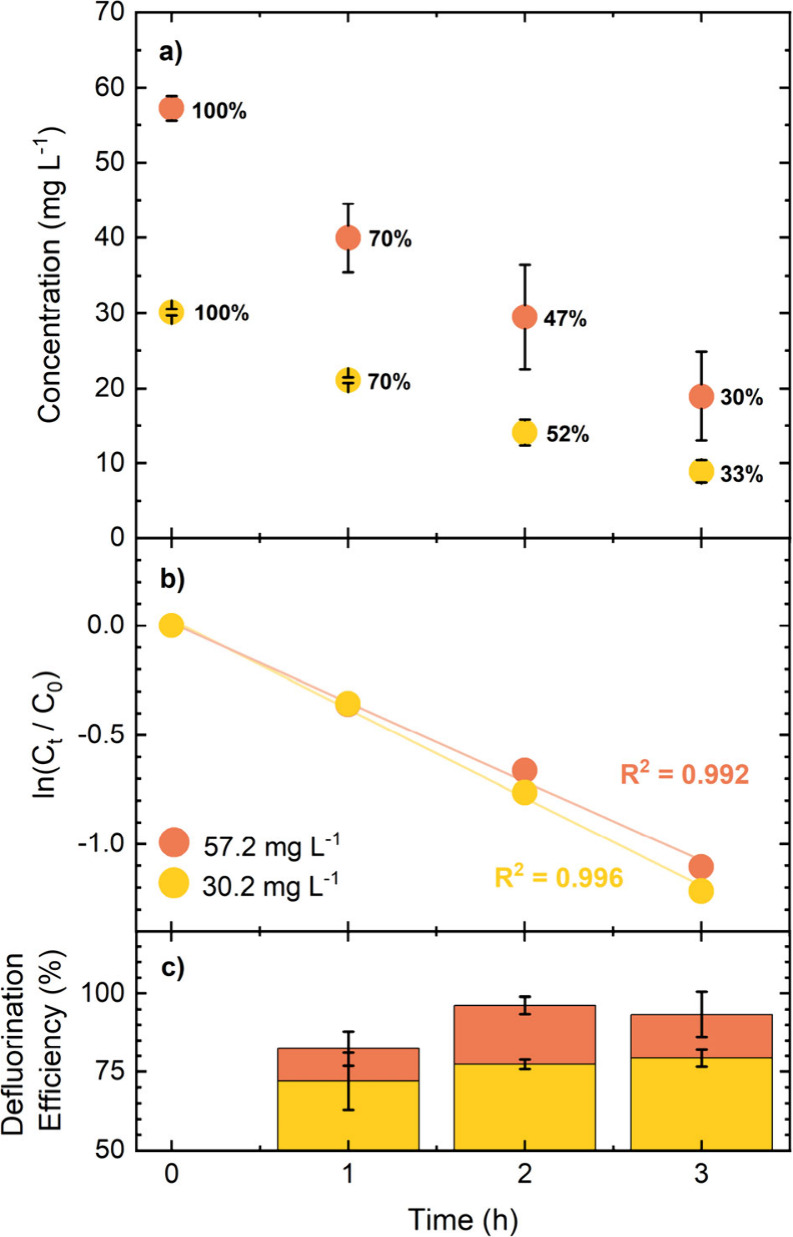
(a) Degradation concentration–time data for PFBS starting at 57.2 mg L^–1^ (orange circles) and 30.2 mg L^–1^ ppm (yellow circles) in saturated K_2_SO_4_ solution. The error bars represent the standard deviation (*n* = 3). Current density = 390 and 480 mA cm^–2^ for outer and inner electrodes respectively; solution volume = 250 mL; total anode area, ∼3.36 cm^2^ (accounting for surface roughness). Numbers next to circles represent the proportion of the initial concentration remaining at each time point. (b) First order kinetic plot of the degradation in (a). (c) Bar chart showing the defluorination efficiency (mass balance) in % for each time point.

The consensus in the literature appears inconsistent when reporting which species (PFBA/S) is degraded faster by electrochemical oxidation, although different studies use different electrolytes and experimental conditions.^30,46^ For our data, although the difference is small, an increased removal rate constant for PFBS over PFBA has been associated with the higher electronegativity of the sulfonate groups (compared to that of carboxylate). This results in neighbouring carbons being more readily activated for electrophilic addition of ^•^OH.^9^

While it is not possible to measure a contact angle directly on the as-grown material used in this study (due to surface roughness), contact angles were measured on a highly polished area of material grown under the same conditions. This was used to determine the hydrophobicity of the BDD electrode. A droplet (10 μL) of ultra-pure water on the oxygen-terminated surface resulted in an average contact angle of 42° (*n* = 3), indicating a hydrophilic surface. Both sulfonate and carboxylate end groups are hydrophilic, with sulfonate likely to be (slightly) more hydrophilic than carboxylate. The addition of 1–60 mg L^–1^ of PFBA or PFBS to the water reduced the contact angle further, by almost one half, due to a lowering of the surface tension (see Table S6 in section SI.6). There are possible advantages of using hydrophilic BDD electrodes for short chain PFAS removal. Short chain PFAS, being less hydrophobic than their longer chain counterparts, should wet the hydrophilic surface more easily. Hydrophilic surfaces also promote favorable interactions of water and electrolyte ions with the surface which is important for electrochemical generation of the radical species.

The defluorination efficiency, while still high, is slightly lower for PFBS than for PFBA at all timepoints, as shown in Figures 3 and 4. At these concentrations this suggests that the breakdown of the intermediates from PFBS electrochemical oxidation is a slightly slower process than for PFBA, even though removal of the initial starting species is slightly faster. PFBA has been detected as a byproduct during PFBS electrolysis, along with other carbonyl terminated degradation intermediates.^30,47^ As for PFBA, the defluorination efficiency of PFBS is higher when the starting concentration is higher.

### Low Concentration PFBA and PFBS

To study degradation at lower concentrations, electrolysis was run in 0.947 mg L^–1^ PFBA and 0.703 mg L^–1^ PFBS in saturated sulfate electrolyte over 4 h. Figure 5 shows the change in PFBA and PFBS concentrations and resulting increase in F^–^ concentration with time. The PFBA and PFBS concentrations fall to 21% and 59% of their starting concentrations respectively over the 4 h electrolysis. The concentration data in Figure 5 were analyzed in terms of eq 1, and first order rate constants for 0.947 mg L^–1^ PFBA and 0.703 mg L^–1^ PFBS were determined as 0.38 ± 0.03 h^–1^ (*R* ^2^ = 0.978) and 0.13 ± 0.007 h^–1^ (*R* ^2^ = 0.993), respectively.

**Figure 5:**
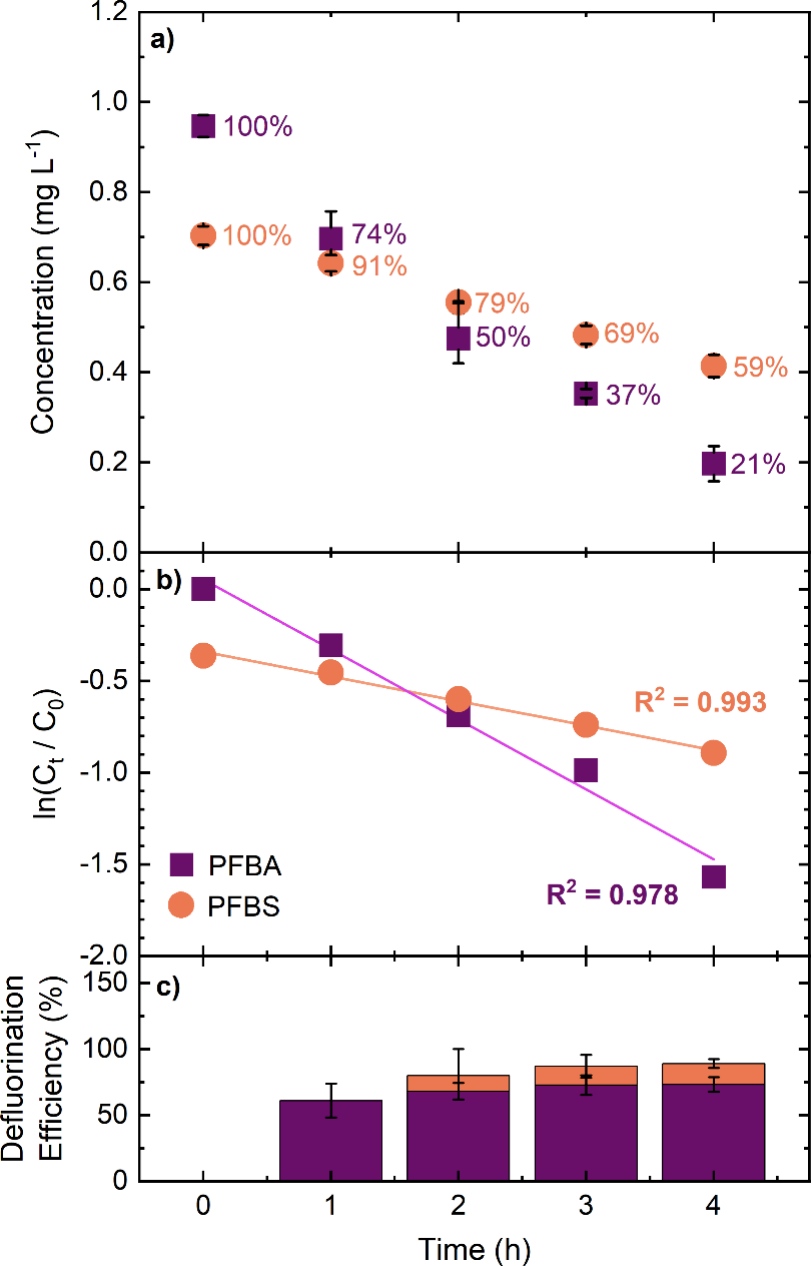
(a) Degradation concentration–time data for PFBA (purple squares) and PFBS (orange circles) starting at 0.947 mg L^–1^ of PFBA and 0.703 mg L^–1^ of PFBS in saturated K_2_SO_4_. The error bars represent the standard deviation (*n* = 3). Current density = 390 and 480 mA cm^–2^ for outer and inner electrodes, respectively; solution volume = 250 mL for PFBA and 0.5 L for PFBS; total anode area, ∼3.36 cm^2^ (accounting for surface roughness). Numbers next to points represent the proportion of the initial concentration remaining at each time point. (b) Pseudo-first-order kinetic plot of the degradation data. (c) Bar chart showing the defluorination efficiency (mass balance) in % for each time point of PFBA and PFBS.

For PFBS at the lowest concentration (for this experiment only), a solution volume of 500 mL was used. To correct for the different volumes used, a treatment volume (*V*)/electrode area (*A*) normalization was applied (*k*_V/A_).^10,32,48^ *k*_V/A_ values are shown in Table 1 for all measurements made. At these low concentrations, the average *k*_V/A_ value for PFBS is now slightly lower than that for PFBA (contrasting with what seen at higher concentration), while the average defluorination % is slightly higher than for PFBA. For both PFBS and PFBA the defluorination % increases with time. A defluorination % value is not provided for time = 1 h (PFBS) due to the amount of F^–^ released being ≤ the limit of detection of the F^–^ ISE. The switch in behavior at low concentration is interesting and is the subject of follow-up work.

**Table 1:** Summary of Experimentally Derived Pseudo First Order *k* Values and Normalized *k*_V/A_ Values for *V* = 250 mL and *A* (Anode) = 3.36 cm^2^ ^a^

compound	*C*_0_ (mg L^‑1^)	*k* (h^‑1^)	*k*_V/A_ (m h^–1^)
PFBA	0.95	0.38 ± 0.03	0.28 ± 0.02
PFBA	19.8	0.29 ± 0.01	0.22 ± 0.01
PFBA	65.7	0.26 ± 0.01	0.19 ± 0.01
PFBS^a^	0.70	0.13 ± 0.007	0.19 ± 0.01
PFBS	30.2	0.41 ± 0.02	0.31 ± 0.01
PFBS	57.2	0.36 ± 0.02	0.27 ± 0.01

a For 0.70 mg L^–1^ PFBS, *V* = 500 mL.

Few studies in the literature have looked at short chain PFBA/PFBS degradation using BDD^30,31,46,49^ and even fewer have provided reaction rate constants and/or defluorination efficiencies.^30,31,46^ In order to compare data from BDD experiments which employ different sized electrodes and treatment volumes, comparison with *k*_V/A_ values is required.^10,32,48^ The results from data in the literature^30,31,46^ are shown in Table S7, section SI.7, for comparison against our results in Table 1. The comparison studies all use lower current densities in magnetically stirred solutions. As Table 1 and Table S7 show, using a higher current density in conjunction with free-standing BDD electrodes, in a recirculating flow cell, has resulted in higher normalized *k*_V/A_ values for PFBA/PFBS degradation, in some cases close to an order of magnitude increase. The area normalizations for comparison literature studies are also made using geometric rather than real surface areas (real surface areas are not given). This means that the actual *k*_V/A_ values for literature values will also be smaller than those provided in Table S7.

In terms of defluorination efficiencies, investigations at BDD electrodes are even more limited.^30,12^ Table 2 lists our defluorination efficiency values as a function of time and PFBA/PFBS concentration; the high values at all time points are evident. For literature comparison, for a solution containing C_3_–C_6_ PFAS at 25 mA cm^–2^ after 1 h, 45% defluorination efficiency was seen (starting PFAS concentrations not stated).^12^ After 1 h for all concentrations in this work defluorination efficiencies are >60%, Table 2. In other literature studies, after 2 h, for a solution containing either 24.4 mg L^–1^ PFBA or 32.4 mg L^–1^ PFBS at 23 mA cm^–2^, defluorination efficiencies of ∼80% and 50% were determined.^30^ Further studies for 20 mg L^–1^ PFBA at 10 mA cm^–2^ after 2 h obtained a defluorination efficiency of 60.8%.^31^ In this work, for similar concentrations of 19.8 mg L^–1^ PFBA and 30.2 mg L^–1^ PFBS at 2 h, we show higher defluorination efficiencies of 94 ± 3% and 77 ± 2%, respectively, Table 2. Our data suggest that the use of a higher current density is more effective at completely breaking down PFBS/PFBA to nonfluorinated compounds when comparing similar time periods/concentrations.

**Table 2:** Defluorination Efficiencies (%) as a Function of Time and PFBA/PFBS Concentration

compound	*C*_0_ (mg L^‑1^)	1 h	2 h	3 h	4 h	9 h
PFBA	0.95	61 ± 12	68 ± 6	73 ± 7	73 ± 6	80 ± 19
PFBA	19.8	86 ± 3	94 ± 3	95 ± 3	n/a	n/a
PFBA	65.7	102 ± 6	106 ± 4	103 ± 2	n/a	n/a
PFBS	0.70	not measurable	81 ± 20	87 ± 9	89 ± 3	103 ± 5
PFBS	30.2	72 ± 3	77 ± 2	79 ± 3	n/a	n/a
PFBS	57.2	82 ± 6	96 ± 3	93 ± 7	n/a	n/a

Given the high current densities used, it is also important to understand the energy efficiency, although there is little comparison data using BDD electrodes for short chain PFAS. Values are calculated for both average energy efficiency, the *G* value (g kWh^–1^)^50^ and electrical energy per order (EEO) in (kWh^–1^ m^–3^)^31^ and are given in Tables S8 and S9 in section SI.8. The *G* and EEO values were found to increase from 0.002 to 0.287 g kWh^–1^ and from 240 to 375 kWh m^–3^, respectively, as PFBA concentration increased. For PFBS, *G* values increased from 0.003 to 0.289 g kWh^–1^, while EEO values decreased slightly from 340 to 291 kWh m^–3^ with increasing concentration.

### Electrode Stability and Repeatability

For the full range of tests carried out, all, bar one experiment (PFBS 0.703 mg L^–1^), employed the same electrochemical cell and BDD electrodes. As a result, the electrodes have been used for over 90 h at ≥390 mA cm^–2^ in high pH solutions (section SI.4). This time includes all the experiments detailed in this paper and additional time spent running the cell at the same current density during set-up experiments prior to paper data collection. While a full study quantifying corrosion of the BDD anode in the presence of PFAS is out of scope for this paper, SEM imaging revealed no discernible differences between regions of the anode that are protected from solution by the O-ring seal (Figure 6a–c) and regions in the middle of the outer anode that have been subjected to a current density of 390 mA cm^–2^ for over 90 h (Figure 6d–f). This suggests that anodic corrosion of the BDD is limited even under these high current density/high pH conditions.

**Figure 6:**
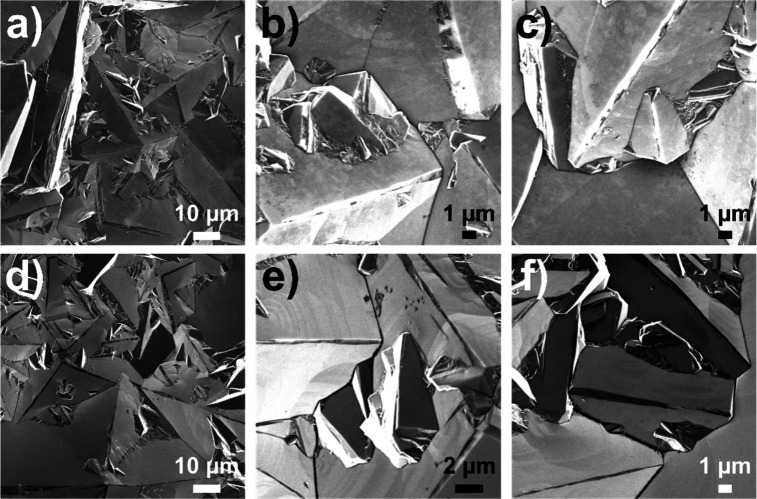
SEM of the outer anode used for all experiments, showing (a–c) the edge of the electrode which is kept dry by the O-ring seal and (d–f) the middle of the anode, which has been subjected to 390 mA cm^–2^ for over 90 h.

The data in the main paper were all carried out at Surrey University (School of Chemistry and Chemical Engineering), U.K. For reproducibility tests, the same electrochemical cell and operating conditions were run by researchers at the University of Warwick (Department of Chemistry) using the ∼20 mg L^–1^ PFBA, saturated K_2_SO_4_ solution (section SI.8). For ease of comparison, only defluorination measurements were made. Figure S7 shows close agreement between the two separate experiments recorded in two separate universities. This cell was also used to record the data in Figure 5 for 0.703 mg L^–1^ of PFBS.

## Conclusions

The use of high current density (≥390 mA cm^–2^, accounting for surface roughness) in combination with free-standing BDD electrodes in a bipolar recirculating flow cell was shown to be highly effective for the destruction of short chain (0.7–66 mg L^–1^) PFBA and PFBS in saturated sulfate solutions, over a 3–9 h time period. First order removal rate constants (when normalized with respect to treatment volume/anode area) in some cases are close to an order of magnitude greater than those in the literature for thin film BDD electrodes operating at lower current densities in stirred solutions. Comparisons have not taken into account differences in electrolyte concentration and identity. Defluorination efficiencies are also higher than all previous BDD electrochemical oxidation literature; in some cases complete defluorination at individual time points is indicated (especially for longer times and for higher PFBA/PFBS concentrations). Such results are likely to be a combination of the material properties of the BDD anode, the high current densities employed, and the flow cell configuration such that the majority of PFAS molecules reaching the electrode surface, per unit time, undergo complete mineralization. Post microscopy analysis of the outer free-standing anode, >90 h of high current density use (in high pH solutions) indicated no obvious signs of corrosion, which is promising for long-term deployment in the field. However, more work is needed to quantify rates of corrosion at the microscopic level^15^ in order to provide reasonable estimates of electrode lifetime.

While the improvements in degradation rate and defluorination efficiency have been shown, the interplay between current density and mass transfer in the flow cell warrants further work. Mass transfer is complex in this flow cell due to the generation of gas bubbles, which are moved away quickly from the electrode surface. Bubble generation could aid in increasing mass transfer in the flow cell via the introduction of turbulence, enabling higher current densities to be employed before the system mass transfer limits. However, its role in blocking electrode sites cannot be discounted. An understanding of the optimal current density in relation to electrochemical hydrostatic flow cell design will be made in future work. This will also aid optimization of electrochemical cell energy efficiency.

This work has considered only oxidative processes at the BDD anodes; however, there is literature which highlights the role of reductive processes at non-BDD electrodes.^51^ Future work will look to investigate whether reductive pathways exist for PFAS breakdown using BDD. Finally, it will be important to consider this approach for use in real systems. Here, other organic content such as that found in landfill leachate, fire-fighting foams, or activated carbon in a drinking water treatment train, etc. will also be present. Operating at high current density could be advantageous in these situations.

## Supplementary Material



## Data Availability

Raw data is available at https://wrap.warwick.ac.uk/192375/.

## Notes

(1) BDD materials characterization; (2) LC-MS/MS method; (3) fluoride ion selective electrode (ISE) measurement method; (4) pH change during electrolysis; (5) gas evolution; (6) contact angle measurements; (7) experimental condition normalized rate constants; (8) energy efficiency, *G*_value_, and EEO values; (9) reproducibility testing between universities (PDF)
