# Supplementary material for: Investigation of Short Chain PFAS Degradation Efficiency Using Free-Standing Boron Doped Diamond Electrodes at High Current Density in a Flow Cell
Source: ACS Electrochem. 2025 Aug 12;1(10):2014–23. doi: 10.1021/acselectrochem.5c00121 (PMC12498407; doi:10.1021/acselectrochem.5c00121)
Supplement: Supplementary file 1 [file ec5c00121_si_001.pdf]

Investigation of Short Chain PFAS Degradation Efficiency Using Free-standing Boron Doped Diamond Electrodes at High Current Density in a Flow Cell

Marius Amerio-Cox,[a] Joshua J. Tully,[b] Fengping Tang,[b] Anna Dettlaff,[c] Julie V. Macpherson,[b]* Timothy Mollart,[d] Tim Sidnell,[a] Simone Mathias,[a] Patrick Sears,[a] Madeleine J. Bussemaker*[a]

[a] School of Chemistry and Chemical Engineering, University of Surrey, GU2 7XH

[b] Department of Chemistry, University of Warwick, Gibbet Hill Road, CV4 7AL, UK

[c]Faculty of Chemistry, Gdańsk University of Technology, 11/12 Gabriela Narutowicza Street, 80-233 Gdańsk, Poland

[d]Element Six (UK) Limited, Oxford, OX11 0QR, UK

Corresponding authors: *j.macpherson@warwick.ac.uk and *m.bussemaker@surrey.ac.uk

SI.1: BDD Material Characterization

SI.2: LC-MS/MS Method

SI.3: Fluoride Ion Selective Electrode (ISE) Measurement Method

SI.4: pH Change During Electrolysis

SI. 5: Gas Evolution

SI.6 Contact Angle Measurements

SI.7 Experimental Condition Normalised Rate Constants

SI.8 Energy efficiency, G_{value},and EEO values

SI.9: Reproducibility Testing Between Universities

SI.1 BDD Material Characterization

(a) Estimation of true anode surface area

Two methods were used to estimate the true surface area of the as-grown BDD material used in this work. The first method used white light interferometry (WLI) to image the electrode surface in 3D. Using a Burker Contour GT the surface was imaged at 10× magnification (Figure S1a) and 20× magnification (Figure S1b). From such images the projected and surface areas can be measured to estimate the area enhancement over a geometric area, due to surface roughness. Figure S1b give an area enhancement of 1.85 for 10× magnification. The second method involved determining the electrochemical surface area of the BDD anode growth surface using the electrical double layer (EDL) capacitance of two “as-grown” electrochemical processing (EP) grade electrodes. The two electrodes, grown to the same thickness as those used in this study, were measured using the procedure described in 1. The disk electrodes had a geometric diameter of 1 mm exposed to solution. The EDL capacitance of the as-grown BDD was compared to that for a 1 mm disk of EP grade BDD material polished flat where the geometric area is now equal to the surface area. An enhancement in the EDL capacitance of ~2.2 was obtained, indicative of a surface area increase of ~2.2 for as-grown over polished. Taking into account these two values, an area enhancement of ~ 2.0 was used when calculating the current densities in the main text.

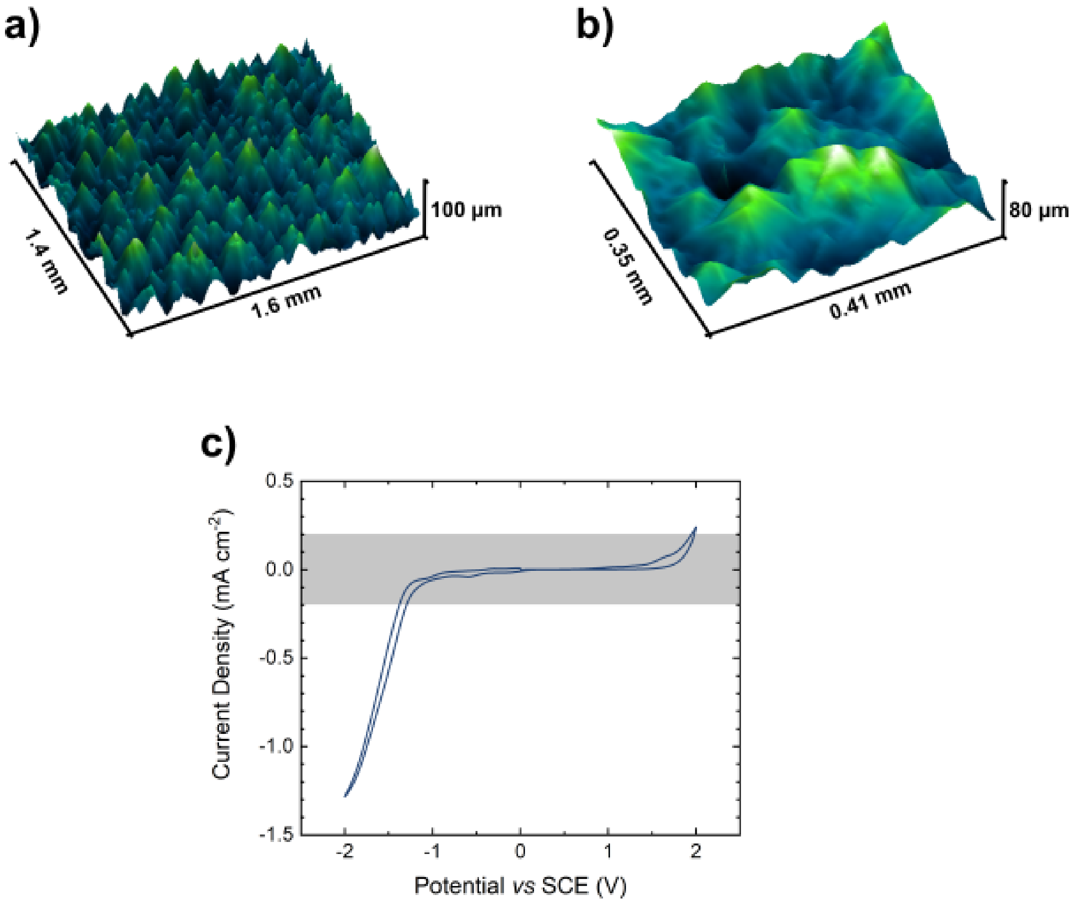

Figure S1: (a) 3D representation of the WLI data collected from the as-grown BDD at 10× magnification. (b) 3D representation of the WLI data collected from the as-grown BDD at 20× magnification. (c) Solvent window of the EP-BDD material used as the anode in these experiments. The grey box indicates the 0.2 A cm^{-2} threshold used to calculate the solvent window.

(b) Electrochemical and Raman Analysis

Measurements were undertaken to characterize the as-grown face of EP grade BDD, which functions as the anode. Electrochemical measurements were conducted using a 302N potentiostat (Autolab, Metrohm, Netherlands) with a SCE reference electrode (CH Instruments, USA) and a platinum wire as a counter electrode. Electrochemical measurements were made on a 10 mm geometric diameter BDD disk, sealed to 8 mm diameter with an O-ring. To measure the solvent window a cyclic voltammogram was measured starting at 0.0 V running positive to 2.0 V and then back to -2.0 V at a scan rate of 100 mV s^{-1} in 0.1 M KNO_3 (Figure S1c). Here, the solvent window is determined as the potential window between which a current density of 0.2 A cm^{-2} (using estimated surface area) is passed in both the oxidative

and reductive windows, as indicated by the grey box in Figure S1c. The solvent window is ~3.2 V, which indicates low sp^2 carbon in the electrode surface.

Raman measurements were also conducted on the same material. Figure S2 shows Raman spectra collected at five random points. These spectra were collected using a Horiba LabRam HR spectrometer with a 488 nm laser at 100% (nominally 50 mW) power. A 600 l/mm grating was used as well as a ×50LWD objective. All spectra show a diamond zone center optical phonon peak, attributed to sp^3 carbon bonding at 1327 cm^{-1}, slightly shifted from 1332 cm^{-1} (intrinsic diamond) due to the incorporation of boron. Asymmetric deformation and attenuation of this peak are visible for all spectra. This feature is attributed to a Fano-type interference between the discrete zone center optical phonon and a continuum of electronic excitations, typically seen when the electrode is doped above the metallic threshold.[2] The higher the doping the greater the asymmetry. As expected for this polycrystalline material, the boron concentration varies across and through the electrode. Non-diamond carbon e.g. graphite and amorphous carbon, is typically seen in polycrystalline BDD between 1400 and 1600 cm^{-1},[3] which also varies heterogeneously across the electrode, with some areas showing negligible

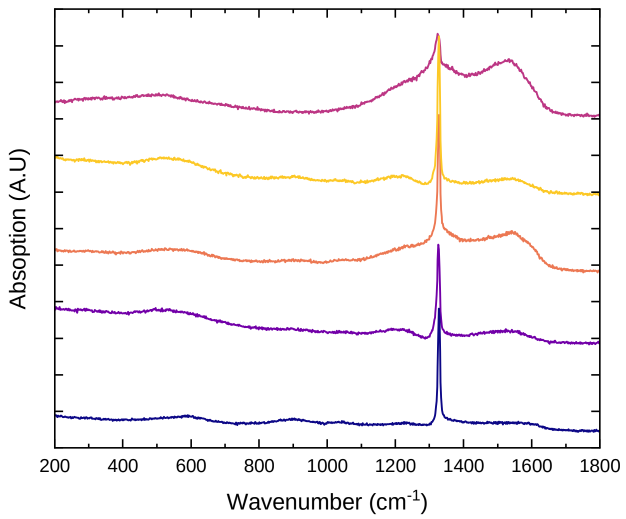

levels.

Figure S2: Five representative Raman spectra from random areas on the EP grade-BDD surface showing the heterogeneity in the material quality for the as-grown polycrystalline surface. The spectra have been offset on the *y*-axis to make visualization clearer.

SI.2: LC-MS/MS Method

The following method was developed for the LC-MS/MS analysis of PFBA and PFBS. n-perfluoroheptanoic acid (PFHpA) (98% Merck) was spiked into the standards and sample solutions at a concentration in the lower third of the calibration curve to act as an internal standard.

Column: Waters BEH C18 1.7 µm, 2.1 × 100mm
Flow Rate: 0.35 ml/min
Mobile Phase A: 20 mM ammonium acetate in water
Mobile Phase B: Methanol
Diluent: Saturated K_2SO_4 solution in water
Weak Wash: 75:25 water: methanol (500 µl)
Strong Wash: 50:50 water: methanol (500 µl)
Seal Wash: 5 min
Column Temp: 45°C
Injection Vol: 10 µl

Table S1: Gradient elution for LC separation of PFAS

**Time (min)**	%B	Curve
**Initial**	10	-
**1.00**	10	6
**2.00**	55	6
**5.25**	85	8
**5.50**	10	6
**6.50**	10	6

Following each injection, a 3.5-min post method was carried out which constituted three wash injections. This was necessary to reduce carryover of PFAS due to the high concentration of the samples tested and reduce salt build up from the high concentration of the K_2SO_4 electrolyte employed. The series of injections were diverted directly to waste and were as follows:

Injection 1: 10 µl of HPLC grade water.

Table S2: Gradient elution for Post Injection 1.

Time (min)	%B	Curve
**Initial**	10	-
**0.75**	10	6
**1.00**	90	6

Injection 2: 10 µl of acetone.

Table S3: Isocratic elution for Post Injection 2.

Time (min)	%B	Curve
**Initial**	90	-
**1.00**	90	6

Injection 3: 10 µl of acetone.

Table S4: Gradient elution for Post Injection 3.

Time (min)	%B	Curve
**Initial**	90	-
**0.75**	10	6
**1.50**	10	6

Electrospray ionisation mass spectrometry method:

Ionisation mode:	ESI negative
Capillary Voltage:	-0.5 kV
Extractor:	-3 V
Source temp	150°C
Desolvation Temp:	400°C
Desolvation gas flow:	700 L/h
Cone gas flow:	0 L/hr
Collision gas:	Argon 3-4 × 10^{-3} mBar

Table S5: Targeted multiple reaction monitoring (MRM) for PFBA, PFBS and n-PFHpA. MRM transitions (bolded) were the most abundant species so were used for quantification of the target analytes and internal standard.

**Compound**	Relative Retention Time to n-PFHpA	MRM Transitions	Cone Voltage (V)	Collision Energy (V)
**PFBA**	0.59	213>**169**	20	10
**PFBS**	0.69	299>**80**<br>299>99	60<br>60	30<br>30
**n-PFHpA**	1.0	363>169<br>363>**319**	15<br>15	15<br>15

The LC column was equilibrated at starting conditions for at least 15 min and an equilibration blank was run before the start of the analysis to run the method gradient through the column prior to the start of the analysis. A seven-point calibration curve was constructed for each experiment, with each data point taken in triplicate, for the quantitative analysis of the targeted PFAS. Figure S3 shows an example calibration curve for the LC-MS analysis of PFBA using n-PFHpA as an internal standard. Samples were then measured in singlet. Responses were ratioed with those of the internal standard. A blank was run between every six samples or standard injections. The calibration curves cover a wide concentration range, which leads to a non-linear response and shows a loss of sensitivity as the detector approaches the limit of detection.

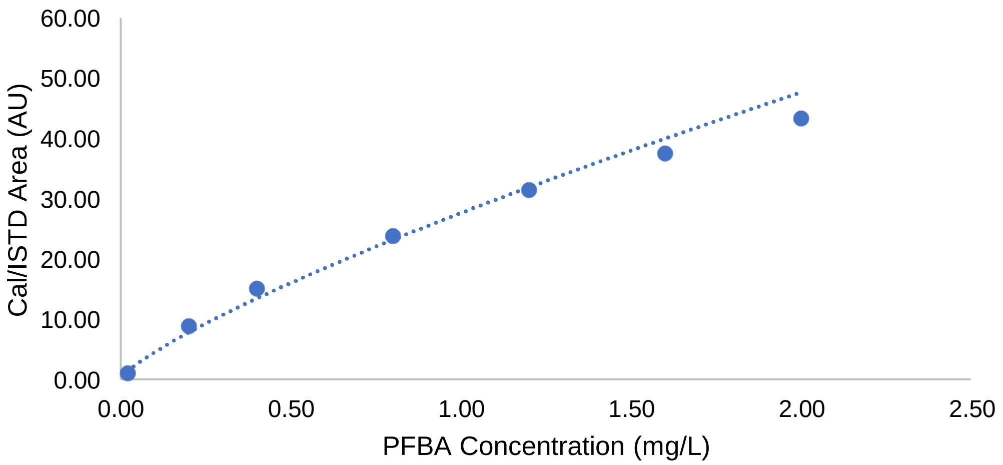

Figure S3: Example calibration curve for the LC-MS/MS analysis of PFBA using n-PFHpA as an internal standard ($y = 27.606x^{0.7867}$, $R^2 = 0.9937$). Error bars representing the standard

deviation at $n = 3$ are too small to be visible on this scale (arising due to the repeatability of the UPLC injection and the use of an internal standard).

SI.3: Fluoride Ion Selective Electrode (ISE) Measurement Method

(F^-) standards were prepared in Milli-Q water over the concentration range 1×10^{-2} M to 1×10^{-6} M each one decade apart. To each standard solution (5 mL total volume), 0.5 mL of ionic strength adjuster buffer (TISAB) was added (Cole-Palmer, Fuoride ISA, Tisab 1). PFAS sample solutions were diluted in a 1:1 ratio with TISAB (pH 4) to ensure they were within the F^- ISE probe operational range.

The F^- ISE and temperature probes were inserted into a 5 mL beaker containing the F^- solution and a stirrer bar was added at 100 rpm. When the F^- ISE voltage remained stable (± 0.05 mV) the temperature was gently increased to 25 ± 0.05°C using a hot plate and the measurement was recorded. Between each sample the probes, beakers and stirrer bar were washed with Milli-Q water and dried. The probe was calibrated and the calibration curve measured in triplicate and samples were measured in singlet, Figure S4.

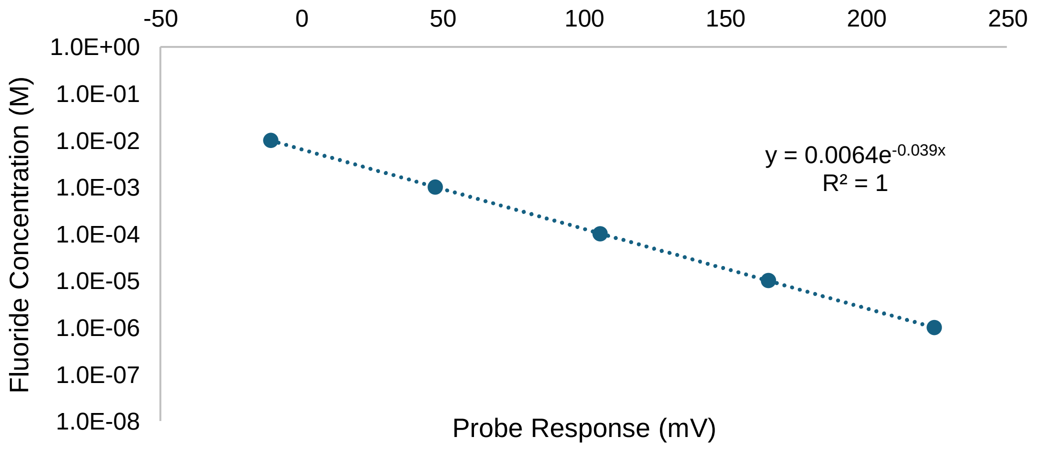

Figure S4: Example calibration curve for the F^- ISE probe analysis ($y = 0.0064e^{-0.039x}$, $R^2 = 1$). Error bars represent standard deviation at *n* = 3.

SI.4 pH Change During Electrolysis

The average pH of the solution throughout the experiment was monitored for the solution containing 19.8 mg L^{-1} PFBA in saturated K_2SO_4, Figure S5. The starting solution pH is 5.6, which rises rapidly to pH 11 in the first 15 mins, then continues to climb to ~ pH 12 after 3 h, remaining approximately constant at this value as the electrolysis period is extended (5 h total). While the cathode produces hydroxide ions *via* the water reduction reaction, production of protons at the anode is occurring *via* the water oxidation reaction. The high pH indicates this process is not balanced. A likely explanation is that a sizeable proportion of the current passing through the anode goes to sulfate oxidation, given the high (saturated) sulfate solution conditions, this process does not produce protons.[4]

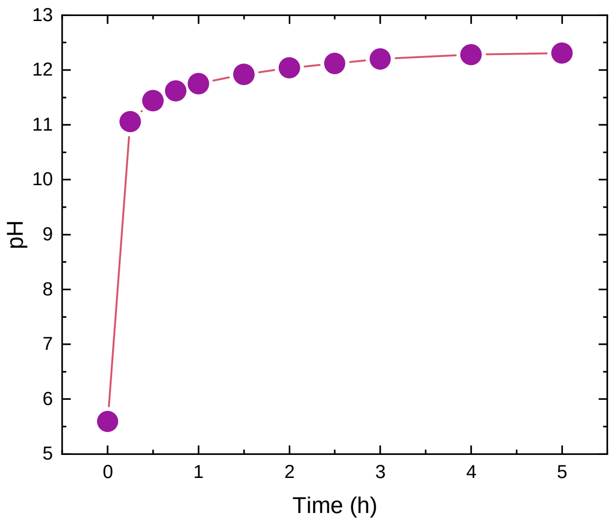

Figure S5: pH of the bulk electrolyte throughout the electrolysis of 19.8 mg L^{-1} PFBA in saturated K_2SO_4.

SI.5 Gas Evolution

Throughout all electrochemical oxidation experiments, significant gas evolution was observed in the outflow pipe (which combines cathode and anode streams), which usually appeared white due to the volume of bubbles. Figure S6 shows the “white” outflow (top) pipe during a typical PFAS electrochemical oxidation experiment. The bottom pipe is the inflow pipe.

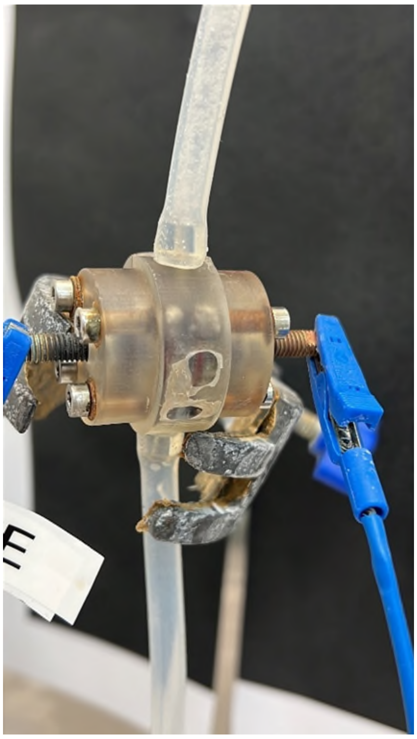

Figure S6: Photograph showing the cell in operation. Note the outflow pipe (top) is much whiter than the inlet pipe (bottom) due to gas evolution occurring within the cell and the bubbles being forced into the outflow pipe.

SI.6: Contact Angle Measurements

Contact angles were measured for the mechanically polished surface of EP grade BDD using a KRÜSS Drop Shape Analysis System (DSA100). A 1 ml syringe was filled with the solution of interest, then positioned above the BDD surface and a 10 µL drop dropped onto the surface and the contact angle measured (*n* = 3). Between solutions the electrode was cleaned by rinsing with methanol, and then ultra-pure water. The results are summarised in Table S6. Concentrations were not quantified using LC-MS and thus are approximations.

Table S6: Contact angle measurements on EP-BDD in several different solutions.

Solution	PFAS	Concentration (mg L^{-1})	Contact Angle
DI Water	None	0	42.07 ± 0.33°
	PFBA	1	26.40 ± 0.51°
		20	26.78 ± 0.06°
		60	22.02 ± 0.02°
	PFBS	1	26.07 ± 0.09°
		20	19.85 ± 0.22°
		60	19.92 ± 0.14°
Saturated K_2SO_4	PFBA	1	14.71 ± 0.37°
		20	17.58 ± 0.31°
		60	20.78 ± 0.26°
	PFBS	1	20.72 ± 0.96°
		20	18.22 ± 0.78°
		60	18.85 ± 0.04°

SI.7 Experimental Condition Normalised Rate Constants

Table S7: Comparison of normalised rate constants with literature values

Paper	PFAS	C_0 (mg L^{-1})	k (h^{-1})	V/A (m)	$k_{V/A}$ (m h^{-1})
Ref [5]; *i* = 0.2 A; anode area = 8.5 cm^2; current density = 23 mA cm^{-2}; volume = 0.04 L	PFBA	24.4	1.99	0.0471	0.094
	PFBS	34.2	0.83	0.0471	0.039
Ref [6]; *i* = 0.3 A; anode area = 3 cm^2; current density = 100 mA cm^{-2}; volume = 0.1 L	PFBA	0.0032	0.054	0.333	0.018
	PFBS	0.0028	0.114	0.333	0.038
Ref [7]; *i* = 0.165 A; anode area = 16.5 cm^2; current density = 10 mA cm^{-2}; volume = 0.1 L (estimate based on 0.25 L cell used)	PFBA	20	0.488	0.061	0.030
	PFBA	2	0.670	0.061	0.041

V is the total volume of the solution and *A* is area of the anode. Note for the normalisations, geometric surface areas are used which will underestimate the real surface area and thus result in a larger $k_{V/A}$ value than reality.

SI.8 Energy efficiency, G_{value} and EEO values

The G_{value} was calculated using eq. S1;[8]

$$G_{value} = \frac{V\ (PFAS_0 - PFAS_t)}{P\ t} \qquad \text{(S1)}$$

where, V is the treatment volume (m^3), P is the average drawn power by the cell (kW), t is the electrolysis time (h), and $PFAS_o$ and $PFAS_t$ are the concentrations (in g / m^3) at time = 0 and t = t.

Under first order reaction conditions, EEO was calculated using eqs. S2 and S3;[9]

$$EEO = \frac{P\ t}{V \log Ci/Cf} \qquad \text{(S2)}$$

where P is the power drawn by the cell (kW), V is the treatment volume (m^3), C_i and $C_{t=0.9}$ are the concentrations of PFAS (mg / L), at time, t (h) = 0 and when 90% degradation of the PFAS (in terms of starting concentration) has been achieved, given by eq. S3.[9]

$$t\,(90\%) = \frac{2.303\ 5851}{k} \qquad \text{(S3)}$$

Table S8: Average results for the electrolysis experiments of PFBA at n = 3.

**$[PFBA]_o$ (mg/L)**	**Electrolysis Time (h)**	**Voltage (V)**	**G_{Value} (g kWh^{-1})**	**EEO (kWh m^{-3})**
65.7	3.0	21±1	0.287	375
19.8	3.0	20±1	0.102	324
0.947	9.0	20±1	0.002	240

Table S9: Average results for the electrolysis experiments of PFBS at *n* = 3.

**$[PFBS]_o$ (mg/L)**	**Electrolysis Time (h)**	**Voltage (V)**	**G_{Value} (g kWh^{-1})**	**EEO (kWh m^{-3})**
57.3	3.0	23±2	0.289	291
27.7	3.0	22±1	0.162	248
0.70	9.0	19±1	0.003	340

SI.9: Reproducibility Testing Between Universities

To investigate the reproducibility of this experimental set-up between laboratories and researchers, electrolysis of a solution containing 20 mg L^{-1} PFBA in saturated K_2SO_4 solution was also run at the University of Warwick by different researchers to those at Surrey University. LC-MS was not used to quantify the initial starting concentration once the solution had been made up to ~20 mg L^{-1}. The cell and BDD electrodes were remade to the exact same design as used by Surrey University. The BDD electrodes were EP Grade BDD (Diafilm™, Element Six) as for all measurements in the main text. Electrodes were cut from the same wafer of EP-BDD as those used by Surrey University.

The experiment was run identically to those run at Surrey University; except the experiment was run for 5 h not 3 h and a treatment volume of 500 mL employed. The solution was circulated using a peristaltic pump (VerderFlex, Vantage 3000 P EZ, Verder, USA). Power was provided by a digital DC power supply (EA-PS 9750-04, Elektro-Automatik GmbH, Germany) and the solution was held at 50 °C in a temperature-controlled water bath (Grant T100, Cambridge, UK). At each time interval a 3.5 mL aliquot of solution was taken, and 3.5 mL of total ionic strength adjustment buffer (TISAB) added to ensure the pH was within the operational range of the F^- ISE (Metrohm, Switzerland). Quantitative MS analysis was not run, but the concentrations of F^- released can be compared to monitor the breakdown of PFBA. The F^- ISE was calibrated in solutions of NaF from 5×10^{-2} M to 2×10^{-6} M in ultrapure water, with an equal volume (3.5 mL) of TISAB added before measurement. The open circuit potential (OCP) was measured three times versus a 3M Ag|AgCl reference electrode (Metrohm, Switzerland) in each solution using a PGSTAT204 potentiostat (Metrohm, Switzerland). A calibration plot of OCP (mV) *vs* -log(F^-), gives excellent linearity over the range investigated ($R^2 = 0.9995$) with a slope of $0.0621 \pm 7.9 \times 10^{-4}$. A comparison of the University of Warwick data and Surrey University data is presented in Figure S7.

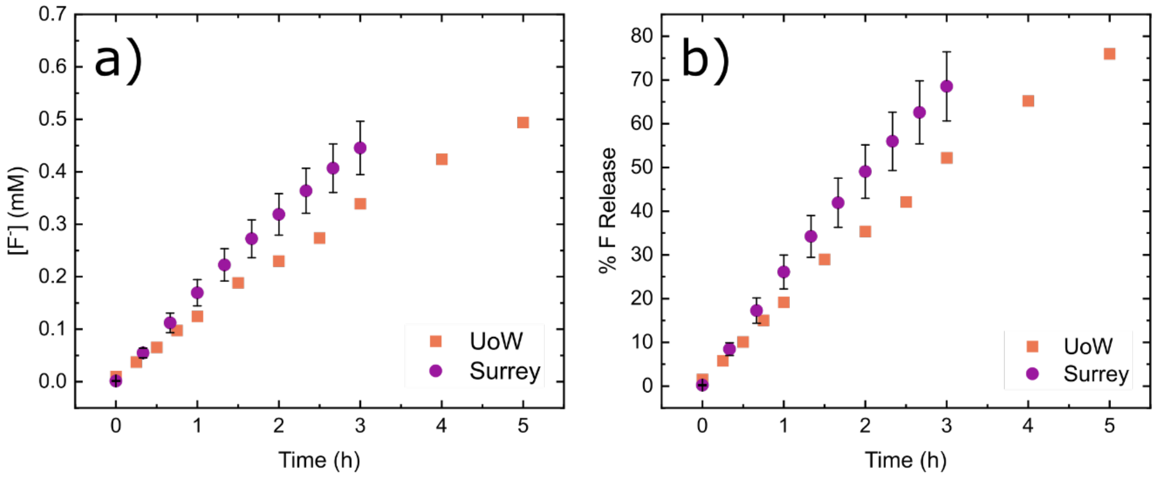

Figure S7: Comparison between F^- ISE data collected at University of Warwick (*), n = 1, and University of Surrey (*), n =3, for electrolysis of ~20 mg L^{-1} PFBA in saturated K_2SO_4. (a) The F^- concentration at different timepoints throughout the experiment. (b) The % of the total fluoride released at different time points based on a maximum fluoride concentration of 0.65 mM when 100% of C-F bonds in the 20 ppm PFBA are broken.

This data shows the both the [F^-] and the %F^- released generally agree between experiments with a continuous rise from t = 0. The data from University of Warwick shows a slightly reduced breakdown rate which could be due to a number of factors, e.g. use of a new BDD electrode, n = 1, slight difference in starting concentrations, double the treatment volume *etc*.

REFERENCES

(1) Hutton, L. A.; Iacobini, J. G.; Bitziou, E.; Channon, R. B.; Newton, M. E.; Macpherson, J. V. Examination of the Factors Affecting the Electrochemical Performance of Oxygen-Terminated Polycrystalline Boron-Doped Diamond Electrodes. *Anal Chem* **2013**, *85* (15), 7230–7240. https://doi.org/10.1021/ac401042t.

(2) Kumar, D.; Chandran, M.; Ramachandra Rao, M. S. Effect of Boron Doping on First-Order Raman Scattering in Superconducting Boron Doped Diamond Films. *Appl Phys Lett* **2017**, *110* (19), 191602. https://doi.org/10.1063/1.4982591.

(3) Liu, Z.; Baluchová, S.; Sartori, A. F.; Li, Z.; Gonzalez-Garcia, Y.; Schreck, M.; Buijnsters, J. G. Heavily Boron-Doped Diamond Grown on Scalable Heteroepitaxial Quasi-Substrates: A Promising Single Crystal Material for Electrochemical Sensing Applications. *Carbon N Y* **2023**, *201* (September 2022), 1229–1240. https://doi.org/10.1016/j.carbon.2022.10.023.

(4) Tully, J. J.; Braxton, E.; Cobb, S. J.; Breeze, B. G.; Markham, M.; Newton, M. E.; Rodriguez, P.; Macpherson, J. V. Diamond Membrane Production: The Critical Role of Radicals in the Non-Contact Electrochemical Etching of Sp2 Carbon. *Carbon N Y* **2021**, *185*, 1–10. https://doi.org/10.1016/j.carbon.2021.09.054.

(5) Zhuo, Q.; Deng, S.; Yang, B.; Huang, J.; Wang, B.; Zhang, T.; Yu, G. Degradation of Perfluorinated Compounds on a Boron-Doped Diamond Electrode. *Electrochim Acta* **2012**, *77*, 17–22. https://doi.org/https://doi.org/10.1016/j.electacta.2012.04.145.

(6) Nienhauser, A. B.; Ersan, M. S.; Lin, Z.; Perreault, F.; Westerhoff, P.; Garcia-Segura, S. Boron-Doped Diamond Electrodes Degrade Short- and Long-Chain per- and Polyfluorinated Alkyl Substances in Real Industrial Wastewaters. *J Environ Chem Eng* **2022**, *10* (2), 107192. https://doi.org/https://doi.org/10.1016/j.jece.2022.107192.

(7) Asadi Zeidabadi, F.; Banayan Esfahani, E.; Moreira, R.; McBeath, S. T.; Foster, J.; Mohseni, M. Structural Dependence of PFAS Oxidation in a Boron Doped Diamond-Electrochemical System. *Environ Res* **2024**, *246*, 118103. https://doi.org/https://doi.org/10.1016/j.envres.2024.118103.

(8) James Wood, R.; Sidnell, T.; Ross, I.; McDonough, J.; Lee, J.; Bussemaker, M. J. Ultrasonic Degradation of Perfluorooctane Sulfonic Acid (PFOS) Correlated with Sonochemical and Sonoluminescence Characterisation. *Ultrason Sonochem* **2020**, *68*, 105196. https://doi.org/10.1016/J.ULTSONCH.2020.105196.

(9) Nzeribe, B. N.; Crimi, M.; Mededovic Thagard, S.; Holsen, T. M. Physico-Chemical Processes for the Treatment of Per- And Polyfluoroalkyl Substances (PFAS): A Review. *Crit Rev Environ Sci Technol* **2019**, *49* (10), 866–915. https://doi.org/10.1080/10643389.2018.1542916.
